# Trends in Abortion Rates in Ontario, Canada

**DOI:** 10.1001/jamanetworkopen.2025.4516

**Published:** 2025-04-11

**Authors:** Laura Schummers, Sheila Dunn, Lucy Cheng, Wendy V. Norman

**Affiliations:** 1 Collaboration for Outcomes Research and Evaluation, Faculty of Pharmaceutical Sciences, The University of British Columbia, Vancouver, British Columbia, Canada; 2 Department of Family and Community Medicine, University of Toronto, Toronto, Ontario, Canada; 3 Women’s College Research Institute, Women’s College Hospital, Toronto, Ontario, Canada; 4 School of Population and Public Health, Faculty of Medicine, The University of British Columbia, Vancouver, British Columbia, Canada; 5 Department of Family Practice, Faculty of Medicine, The University of British Columbia, Vancouver, British Columbia, Canada; 6 Department of Public Health, Environments and Society, Faculty of Public Health and Policy, London School of Hygiene & Tropical Medicine, London, United Kingdom

## Abstract

**Importance:**

Following decades-long declines, significant increases in abortion rates have been reported in some jurisdictions from 2020 to 2023, but it is not yet known whether these trends are occurring in Canada.

**Objective:**

To assess abortion rates in Ontario from 2012 to 2022 and to examine trend changes associated with mifepristone availability, the COVID-19 pandemic, and postpandemic periods.

**Design, Setting, and Participants:**

This population-based interrupted time series cohort study examined all medication and procedural abortions provided in Ontario from January 1, 2012, to December 31, 2022, to females aged 15 to 44 years with provincial insurance coverage, identified using linked health administrative data that included records from practitioner billings, inpatient and outpatient hospital services, same-day surgeries, and outpatient prescription dispensations.

**Exposure:**

Availability of mifepristone regulated as a normal (ie, prescribed by an authorized prescriber without additional certification or registration and dispensed by a pharmacist) prescription medication (in November 2017) and the COVID-19 pandemic period (from March 2020 to December 2021).

**Main Outcomes and Measures:**

The main outcome was the abortion rate (number of abortions per 1000 females per year) overall and within age strata, using an interrupted time series design.

**Results:**

Of 422 867 medication and procedural abortions identified using data from health records of 225 540 reproductive-aged females (mean [SD] age, 28.5 [6.6] years), the abortion rate declined steadily from 15.6 abortions per year per 1000 females, aged 15 to 44 years, in 2012 to 12.3 in 2021 and then increased to 14.1 in 2022. When mifepristone was introduced in 2017 as a normal prescription medication, no immediate change in the abortion rate (−0.1 [95% CI, −0.7 to 0.8]) and a nonsignificant slope increase (0.6 [95% CI, −0.5 to 0.7]) were found. However, this trend resulted in an additional 1.5 (95% CI, 0.3-2.6) abortions per 1000 females by the first quarter of 2020 compared with premifepristone trends; rates increased more among those aged 15 to 19 years, less among those aged 35 to 44 years, and did not increase for those aged 25 to 29 years. During the pandemic period, abortion rates decreased by 1.2 (95% CI, −2.5 to −0.8), most pronounced among those aged 20 to 34 years. Compared with expected rates based on premifepristone trends, 5-year availability of normally prescribed mifepristone was associated with a rate difference of 1.9 (95% CI, 0.7-5.4) in 2022, with a greater increase among those aged 20 to 24 years (4.2 [95% CI, 1.5-9.0]) and no change among those aged 25 to 29 years (1.0 [95% CI, −1.7 to 6.2]). The increased abortion rate in 2022 was consistent with 5-year trends following normally prescribed mifepristone, although social forces potentially impacting international rates may have contributed.

**Conclusions and Relevance:**

This study found that, following longstanding declines, abortion rates in Ontario gradually increased with mifepristone availability in 2017 in Ontario. Following a pandemic-related decrease in rates (in 2020 and 2021), substantial increases in abortion rates reported elsewhere from 2020 to 2023 did not occur in Ontario as of 2022, suggesting that Ontario’s health services environment and Canada’s regulatory and policy approach to preserving reproductive health services may have helped stabilize abortion rates. Future research is needed to understand how sociocultural changes affecting abortion service use elsewhere may be affecting contraception access and use and thus abortion rates in Canada.

## Key Points

**Question:**

How have abortion rates in Ontario, Canada, changed since the introduction of mifepristone in 2017, during the COVID-19 pandemic from 2020 to 2021, and in 2022?

**Findings:**

This cohort study examined changes in abortion rates from 2012 to 2022 in Ontario using a cohort of 422 867 medication and procedural abortions identified from health records of reproductive-aged females. Abortion rates were declining before mifepristone availability (from 2012 to 2016), increased gradually after the introduction of mifepristone, decreased during the pandemic, and then returned to prepandemic trends.

**Meaning:**

The findings that after longstanding declines, abortion rates in Ontario—which gradually increased with mifepristone availability in 2017 and decreased during the COVID-19 pandemic—did not substantially increase from 2020 to 2022, may reflect Canada’s distinct reproductive health policy environment and Ontario’s health services context.

## Teaser

This cohort study examines changes in abortion rates for medication and procedural abortions in Ontario, Canada, from 2012 to 2022, including trend changes associated with the availability of mifepristone, the COVID-19 pandemic, and postpandemic periods.

## Introduction

Following decades-long decreasing abortion rates, substantial increases in rates were reported in the US, UK, and Europe from 2020 to 2023. In Scotland, following a stable rate of 13.0 to 13.5 abortions per 1000 females aged 15 to 44 years per year from 2018 to 2021, rates rose abruptly to 16.1 in 2022 and to 17.6 in 2023 (the highest since abortion surveillance began).^1^ In England and Wales, following a period of stable rates from 2012 to 2016 (approximately 16.0 per 1000 females aged 15 to 44 years), abortion rates rose steadily to 18.2 in 2020 and then increased markedly to 20.6 in 2022 (the highest since the UK Abortion Act 1967 was introduced).^2^ In Switzerland, despite substantially lower baseline rates (6.3 to 6.9 from 2018 to 2021), abortions rose to 7.3 in 2023 (the highest since 2004).^3^ In the US, rates increased from 11.2 in 2020 to 15.9 in 2023^4^ despite state abortion bans reducing access.^5,6^

It is not yet clear whether such trends are occurring in other international jurisdictions, including Canada. In jurisdictions where increasing abortion rates have been found, increases have been associated with decreasing use of effective contraception,^7^ rising influences of contraception misinformation or disinformation on social media,^8,9,10^ cost of living,^11^ collapsing primary care and sexual health services,^12^ and improved abortion access.^4^

In Canada, it remains unknown whether social phenomena potentially increasing abortion rates are occurring or how such phenomena may interact with abortion rate trends resulting from the introduction of mifepristone in January 2017^13^ and subsequent policy changes that removed all mifepristone-specific regulations and health professional training or certification requirements by November 2017.^14^ Under this globally unprecedented policy framework, mifepristone became normally prescribed, which we define as the typical way a prescription (such as penicillin) is managed (ie, any authorized prescriber [physician, nurse practitioner, or midwife] can prescribe mifepristone without additional certification or registration, and any pharmacist can dispense it as a routine prescription medication for patient use). A 2022 study reported that, as of March 2020, normally prescribed mifepristone was associated with a sharp increase in the proportion of abortions provided by medication (from 2.2% in 2017 to 31.4%) and an attenuated decline in the abortion rate (resulting in an additional 1.2 abortions per 1000 females, aged 15-49 years, than expected).^15^

During the COVID-19 pandemic (from March 2020 to December 2021), abortion service use decreased in many jurisdictions,^16,17,18,19^ potentially due to disruptions in service provision^17,18,20^ and/or reduced service need following decreases in sexual activity and pregnancy rates.^17,21,22^ With classification of abortion as an essential service by The Society of Obstetricians and Gynaecologists of Canada^23^ and the increasing provision of medication abortion, Canadian abortion services may have been more resilient to pandemic service disruptions. However, pandemic-related abortion rate trends have not been described in Canada.

In this study, we described abortion rate trends and use of medication abortion from 2012 to 2022 in the province of Ontario, Canada. Using an interrupted time series design, we characterized changes in abortion rates during the COVID-19 pandemic and compared observed abortion rates in 2022 with expected rates based on both premifepristone and postmifepristone prepandemic trends.

## Methods

### Study Population

In this cohort study, we identified a population-based cohort of females aged 15 to 44 years with provincial insurance coverage in Ontario from January 1, 2012, to December 31, 2022, where approximately 40% of Canada’s population lives and approximately 41% of abortions in Canada occur.^24,25^ We identified all medication and procedural abortions provided to individuals in this cohort using linked health administrative data, including records from practitioner billings, inpatient and outpatient hospital services, same-day surgeries, and outpatient prescription dispensations. These datasets were linked using unique encoded identifiers and analyzed at ICES, using an established linkage approach and case definition for procedural and medication abortions (eTable 1 in Supplement 1)^15,26^ based on a previous validation study.^27^ ICES is a prescribed entity under Ontario’s Personal Health Information Protection Act (PHIPA). Section 45 of PHIPA authorizes ICES to collect personal health information, without consent, for the purpose of analysis or compiling statistical information, with respect to the management of, evaluation or monitoring of, the allocation of resources to, or planning for all or part of the health system. Projects that use data collected by ICES under section 45 of PHIPA and use no other data are exempt from research ethics board review. The use of the data in this project is authorized under section 45 and approved by ICES’ Privacy and Legal Office. The study followed the Strengthening the Reporting of Observational Studies in Epidemiology (STROBE) reporting guideline.

### Study Setting

In Canada, following decriminalization in 1988, abortion became regulated as a normal part of health care.^28^ Mifepristone was first introduced in Canada in January 2017.^13^ Initially, regulations governing mifepristone practice mirrored those in the US and elsewhere (eg, prescriber training, patient consent form, direct prescriber dispensing, and observed dosing).^29,30^ From January to November 2017, these restrictions were incrementally removed, resulting in a globally unique regulatory approach in which mifepristone became a normally prescribed medication as of November 7, 2017.^14^ Under this model, abortion service provision spread beyond purpose-specific specialty clinics^31^ to geographically diverse community primary care settings,^32^ including by telemedicine.^33^

### Statistical Analysis

We described annual abortion rates (the number of abortions per year per 1000 females) and medication abortion percentages (percentage of abortions provided by medication) overall and by 5-year age categories. To examine the potential influence of mifepristone introduction and the COVID-19 pandemic on abortion rate trends, we conducted an interrupted time series analysis.^34^ We used quarterly observations to identify abortion rate trends before mifepristone availability (quarter 1 in 2012 to quarter 4 in 2016), after mifepristone availability as a normally prescribed medication but before the COVID-19 pandemic (quarter 4 in 2017 to quarter 1 in 2020), during the COVID-19 pandemic (quarter 2 in 2020 to quarter 4 in 2021), and after the COVID-19 pandemic (quarters 1 to 4 in 2022), when pandemic mitigation measures were generally no longer in effect^35^ (postpandemic), in the overall population and within 5-year age strata (with ages 35 to 44 years collapsed due to small counts). We excluded quarters 1 to 3 in 2017 in our models, as this mifepristone phase-in period included rapid incremental service changes.^29^ We considered linear, quadratic, and log transformations; we selected the transformation that minimized Akaike information criterion and, when appropriate, compared nested models using likelihood ratio tests.^36^ This resulted in log transformations for premifepristone and postmifepristone trend periods in the overall cohort (age-stratum transformations are provided in eTable 2 in Supplement 1). We estimated the change in abortion service use during the COVID-19 period, with pandemic era rates modeled as wild outlier observations.^37^ We compared the observed abortion rate in quarter 4 in 2022 with the expected rate based on premifepristone trends and postmifepristone prepandemic trends, with all trends accounting for quarterly seasonality.^37^ Finally, we estimated the difference between expected rates based on premifepristone trends and expected rates based on postmifepristone prepandemic trends to quantify the 5-year outcome of normally prescribed mifepristone. We estimated rate differences and accompanying 95% CIs using bootstrapping, adjusted for seasonality in abortion rates.^38^ All analyses were conducted using SAS, version 9.4 (SAS Institute Inc) and R, version 4.4.1 (R Project for Statistical Computing). Two-sided *P* values with a threshold of α = .05 was considered statistically significant.

## Results

**Figure 1.**
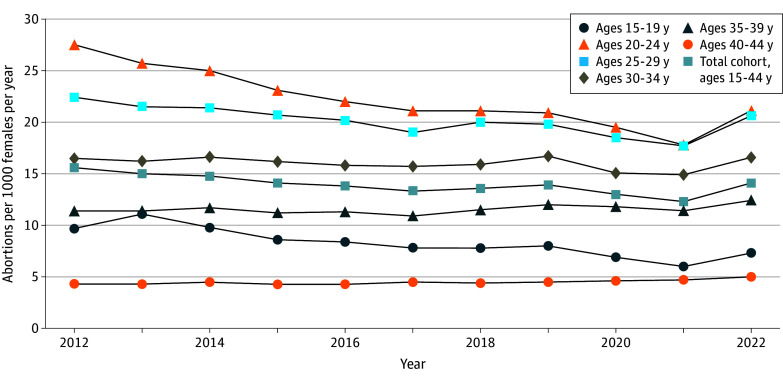
Annual Abortion Rate Trends in Ontario, Canada, 2012 to 2022, Overall and Within 5-Year Age Categories The abortion rate is defined as the number of abortions in each year divided by the number of females aged 15 to 44 years in the population in that year.

We examined 422 867 medication and procedural abortions identified using data from health records of 225 540 reproductive-aged females (mean [SD] age, 28.5 [6.6] years) from January 2012 to December 2022. As shown in Figure 1, the abortion rate declined steadily from 15.6 per year per 1000 females aged 15 to 44 years in 2012 (n = 42 015) to 13.8 in 2016 (n = 37 272), reaching a minimum of 12.3 in 2021 (n = 35 368) and then increasing to 14.1 in 2022 (n = 41 320). In the premifepristone period, relative declines from 2012 to 2016 were greatest for those aged 15 to 24 years, followed by those aged 25 to 29 years; in contrast, abortion rates were fairly stable among those aged 30 to 44 years from 2012 to 2016.

**Figure 2.**
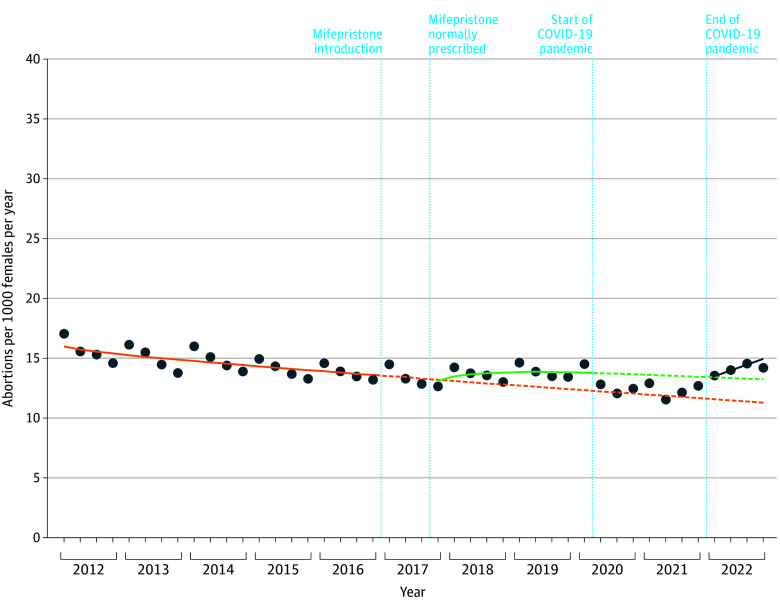
Interrupted Time Series Model Evaluating Changes in the Abortion Rate Among Females Aged 15 to 44 Years in Ontario, Canada The dark blue dots indicate the observed abortion rate (abortions per 1000 females) in each quarter from January 2012 to December 2022. The orange solid curve indicates the observed abortion rate trend from 2012 to 2016; the orange dashed curve indicates the expected trend from January 2017 to December 2022 based on the premifepristone trend. The green solid curve indicates the observed abortion rate trend after mifepristone was available as a normally prescribed medication from November 2017 to March 2020; the green dashed curve indicates the expected trend with normally prescribed mifepristone from April 2020 to December 2022. The blue line indicates the observed trend from January to December 2022.

The results of our interrupted time series analysis are plotted in Figure 2, with rate differences shown in the Table and abortion rates in each period in eTable 3 in Supplement 1. When mifepristone first became available as a normally prescribed medication (quarter 4 in 2017), the abortion rate remained stable, with a small, nonsignificant immediate (level) change of −0.1 (95% CI, −0.7 to 0.8) and a small, nonsignificant slope change (0.6 abortions per year per 1000 females aged 15 to 44 years [95% CI, −0.5 to 0.7]). However, this trend resulted in an additional 1.5 (95% CI, 0.3-2.6) abortions per 1000 females by quarter 1 in 2020, before the COVID-19 pandemic, compared with the expected rate if mifepristone had not been introduced (Figure 2). Rates increased more among those aged 15 to 19 years, less among those aged 35 to 44 years, and did not increase for those aged 25 to 29 years. During the pandemic period (quarter 2 in 2020 to quarter 4 in 2021), the abortion rate decreased by 1.2 (95% CI, −2.5 to −0.8) abortions per 1000 females per year, most pronounced among those aged 20 to 34 years, compared with the expected values based on prepandemic trends.

**Table.** Abortion Rate Differences Associated With Mifepristone Availability as a Normally Prescribed Medication and With the COVID-19 Pandemic, by Age^a^

Age group, y	Mifepristone availability as a normally prescribed medication			COVID-19 era change: April 2020 to December 2021, rate difference (95% CI)	After the COVID-19 pandemic, October to December 2022		
	October to December 2017, immediate (level) change (95% CI)	October to December 2017, slope change (95% CI)	January to March 2020, rate difference (95% CI)		Observed minus expected based on premifepristone trend, rate difference (95% CI)	Observed minus expected based on postmifepristone trend, rate difference (95% CI)	Expected based on premifepristone trend minus expected based on postmifepristone trend, rate difference (95% CI)
15-44 (Total cohort)	−0.1 (−0.7 to 0.8)	0.6 (−0.5 to 0.7)	1.5 (0.3 to 2.6)	−1.2 (−2.5 to −0.8)	2.9 (1.0 to 4.4)	1.0 (−1.9 to 1.3)	1.9 (0.7 to 5.4)
15-19	0.4 (−0.5 to 1.4)	0.4 (−0.6 to 0.9)	1.8 (0.5 to 3.3)	−1.3 (−2.5 to −0.5)	2.6 (2.4 to 6.7)	−0.2 (−0.8 to 3.2)	2.8 (0.6 to 6.4)
20-24	0.9 (−0.6 to 2.1)	0.2 (−0.2 to 0.6)	2.5 (−1.9 to 7.3)	−1.8 (−3.3 to −0.5)	5.7 (1.3 to 11.0)	1.5 (0.1 to 2.7)	4.2 (1.5 to 9.0)
25-29	−0.6 (−1.7 to 0.8)	0.8 (−0.8 to 1.2)	0.8 (−1.0 to 2.8)	−1.7 (−3.5 to −0.8)	2.6 (−0.2 to 5.5)	1.6 (−2.2 to 3.2)	1.0 (−1.7 to 6.2)
30-34	−0.1 (−0.3 to 0.4)	0.3 (0.1 to 0.3)	2.9 (0.6 to 3.2)	−2.5 (−2.7 to −1.9)	5.1 (0.2 to 6.0)	−1.0 (−1.5 to 0)	2.9 (0.6 to 3.2)
35-44	0 (−0.3 to 0.6)	0.4 (−0.4 to 0.4)	1.1 (0.6 to 2.0)	−0.5 (−1.4 to −0.4)	1.8 (0.8 to 3.0)	0.1 (−1.8 to 0.2)	1.7 (1.3 to 4.2)

^a^ Rates used to estimate rate differences were adjusted for seasonality; 95% CIs were estimated using bootstrapping.

After the COVID-19 pandemic (quarters 1 to 4 in 2022), we found that abortion rates were similar to the expected values based on prepandemic trends and seasonal fluctuations observed in all study years. From October to December (quarter 4) 2022, we observed an abortion rate of 14.2 per 1000 females, which was consistent with the expected rate of 13.2 (95% CI, 12.9-16.1), based on postmifepristone trends, with a nonsignificant rate difference of 1.0 abortions (95% CI, −1.9 to 1.3). Comparing the expected rate in October to December 2022, based on premifepristone trends with the expected rate based on postmifepristone trends, we found a 5-year rate difference of 1.9 (95% CI, 0.7-5.4) abortions per year per 1000 females (Figure 2).

**Figure 3.**
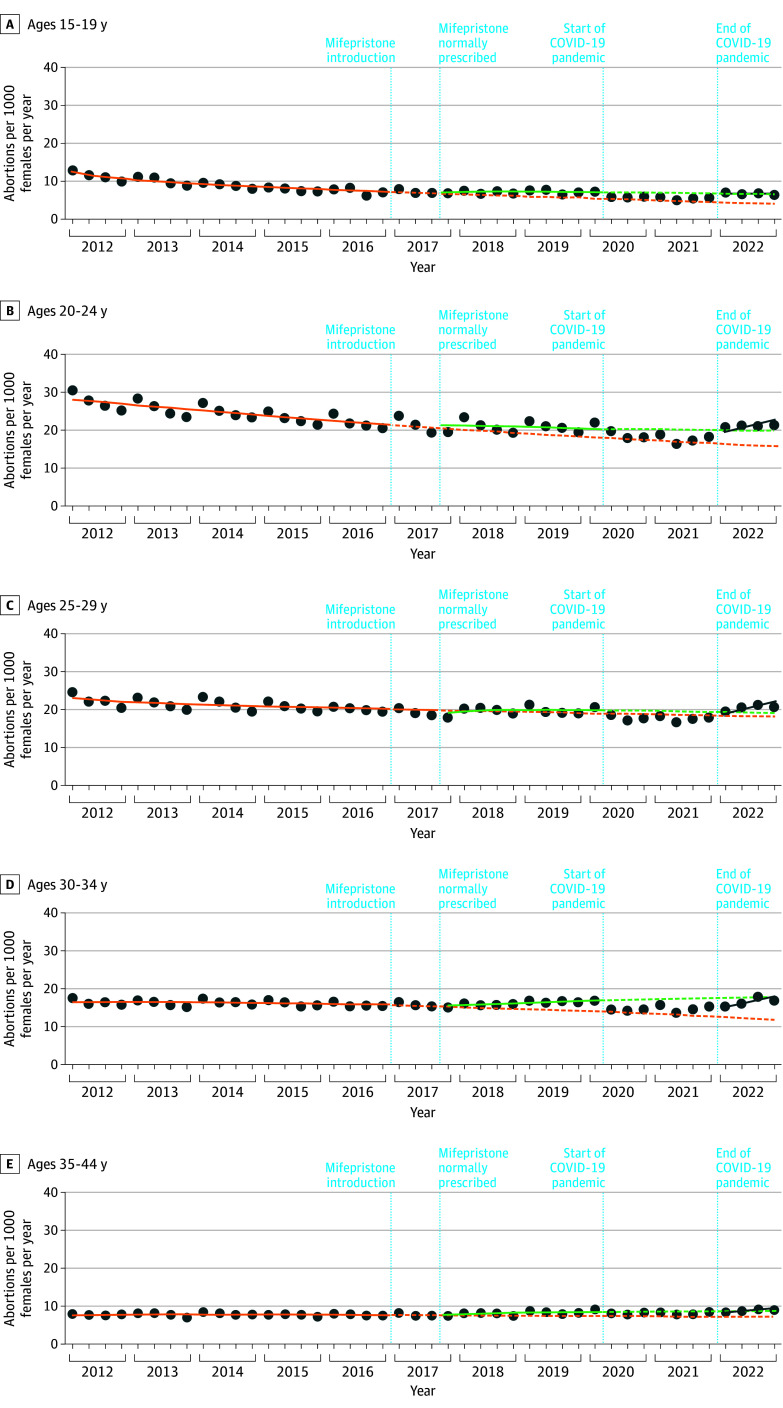
Age-Stratified Interrupted Time Series Model Evaluating Changes in the Abortion Rate in Ontario, Canada Within each age stratum, the dark blue dots indicate the observed abortion rate (abortions per 1000 females) in each quarter from January 2012 to December 2022. The orange solid curves indicate the observed abortion rate trends from 2012 to 2016; the orange dashed curves indicate the expected trends from January 2017 to December 2022 based on premifepristone trends. The green solid curves indicate the observed abortion rate trends after mifepristone was available as a normally prescribed medication from November 2017 to March 2020; the green dashed curves indicate the expected trends with normally prescribed mifepristone from April 2020 to December 2022. The blue lines indicate the observed trends from January to December 2022.

**Figure 4.**
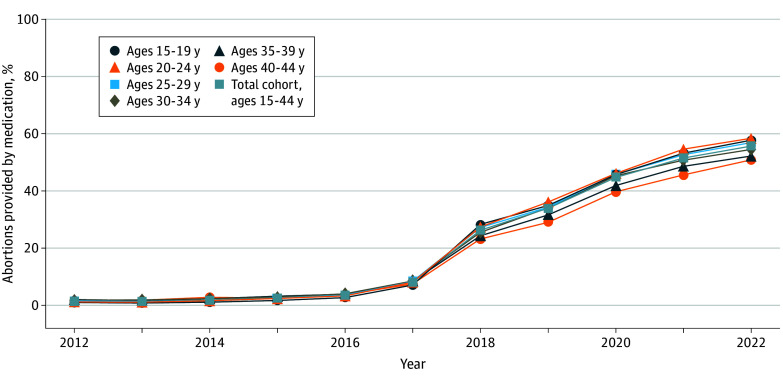
Trends in the Percentage of Abortions Provided by Medication in Ontario, Canada, From 2012 to 2022, Overall and Within 5-Year Age Categories The percentage of abortions provided by medication is defined as the number of medication abortions divided by the number of all identified abortions in each year.

As shown in Figure 3, we found nonsignificant immediate changes and small slope increases in the abortion rate when mifepristone became available as a normally prescribed medication in all age strata (with the exception of a small, significant trend increase of 0.3 [95% CI, 0.1-0.3] abortions per quarter among those aged 30 to 34 years). We found the greatest number of additional abortions as of quarter 1 in 2020 (before the pandemic) among those aged 15 to 19 years (rate difference: 1.8 [95% CI, 0.5-3.3]) and aged 30 to 34 years (rate difference: 2.9 [95% CI, 0.6-3.2]), a smaller increase among those aged 35 to 44 years (rate difference: 1.1 [95% CI, 0.6-2.0]), and nonsignificant changes among those aged 25 to 29 years (rate difference: 0.8 [95% CI, −1.0 to 2.8]) and aged 20 to 24 years (rate difference: 2.5 [95% CI, −1.9 to 7.3]). Abortion rates declined in all age groups during the COVID-19 pandemic, with the largest decreases in those aged 30 to 34 years (rate difference: −2.5 [95% CI, −2.7 to −1.9]) and the smallest change in those aged 35 to 44 years (−0.5 [95% CI, −1.4 to −0.4]). In quarter 4 in 2022, comparing expected values based on premifepristone trends with expected values based on postmifepristone trends, these 5-year rate differences were largest among those aged 20 to 24 years (4.2 [95% CI, 1.5-9.0]), followed by those aged 30 to 34 years (2.9 [95% CI, 0.6-3.2]) and aged 15 to 19 years (2.8 [95% CI, 0.6-6.4]) and were smallest in those aged 35 to 44 years (1.7 [95% CI, 1.3-4.2]), with a nonsignificant change for those aged 25 to 29 years (1.0 [95% CI, −1.7 to 6.2]). In most age strata, the observed rates in 2022 were consistent with expected values based on postmifepristone trends. However, for those aged 20 to 24 years, the observed rate in quarter 4 in 2022 (21.4) was slightly above the expected rate of 19.9 (95% CI, 18.8-21.3). As shown in Figure 4, the percentage of all identified abortions provided by medication abortion continued to increase throughout our post-2017 study period in all age groups from less than 2% in 2012 to 34% in 2019 (as previously reported^15^) to 56% in 2022.

## Discussion

Our population-based cohort study using an interrupted time series design to examine abortion rate trends in Ontario, Canada, from 2012 to 2022 found that longstanding abortion rate declines continued through 2016. In this period, declines were steepest for those younger than 25 years, and rates were stable for those aged 30 to 44 years. Rates increased modestly when mifepristone became available as a normally prescribed medication from 2017 to 2020 and then decreased during the COVID-19 pandemic in 2020 and 2021. The increased abortion rate in 2022 was generally consistent with trends following normally prescribed mifepristone over 5 years, although social forces potentially impacting international rate increases may also have contributed to this increase. Our findings suggest that, after accounting for pandemic-related rate variability, substantial increases in abortion rates reported elsewhere from 2020 to 2023^1,2,3,4^ did not occur in Ontario as of 2022. The timing of Canada’s 2017 introduction of mifepristone and rapid availability as a normally prescribed medication may not have reached postmifepristone equilibrium before the COVID-19 pandemic, or before more recently described sociocultural changes potentially affecting abortion services, occurred. Thus, whether abortion rate trends in Canada will experience abrupt increases over time or will continue to follow expected trends remains uncertain.

Following the introduction of mifepristone as a normally prescribed medication, the abortion rate among those aged 15 to 44 years increased modestly compared with the expected declining trend in the overall population. This finding was consistent with a prior study of this policy change among those aged 15 to 49 years over 2 years after the policy change,^15^ with a small added increase accumulated over 5 years after the policy change. With normally prescribed mifepristone, abortion became increasingly available in primary care^32,39,40^; while some logistic barriers to telemedicine abortion persist,^41^ telemedicine provision is widely used under this policy framework.^33^ Thus, at least some of the increase in the abortion rate in this 5-year period may reflect the magnitude of unmet needs for abortion services under Canada’s previous abortion service model.^42,43^ Variability in rate differences by age over this period may reflect heterogeneity with respect to age-specific premifepristone access barriers, method preference,^44^ and age-specific changes in use of contraception methods,^7^ including age-specific exposure to contraception misinformation or disinformation on social media^8,9,10^ or other sociocultural changes during this period. In most age groups, the increased rates in 2022 were consistent with expected values based on trends with normally prescribed mifepristone; however, for those aged 20 to 24 years, the observed rate was slightly higher than the expected range, which may reflect age-specific influences of social phenomena driving international rate increases.

If sharp increases in the abortion rate from 2020 to 2023 reported in some countries were due to declining use of the most effective contraception methods,^7^ perhaps a result of growing contraception misinformation or disinformation social media campaigns^8,9,10^ or cost-of-living concerns resulting from postpandemic inflation,^11^ these influences may have contributed to the increase in our study’s results in 2022 or may continue to accrue in Canada in the coming years. On the other hand, if these increases were due to reduced access to the most effective contraception^12^ or improved abortion access^4^ (such as through increasing availability of telemedicine and/or self-managed abortion that was first introduced during the pandemic in many countries^45,46,47,48^), Canada’s distinct health services and policy environment may result in distinct trends. Across Canada, several policy changes removing cost-related barriers to contraception were implemented from 2017 to 2024, including Ontario’s 2017 policy that provides free prescription medication for provincially insured residents under age 25 years^49^ (associated with increased contraception use among youth^50^), British Columbia’s 2023 policy that provides free contraception for all provincially insured residents,^51^ and a 2024 national policy that will provide free contraception across the country through bilateral provincial–federal agreements.^52^

Despite continued increases in the percentage of abortions provided by medication, Canada’s percentage of medication abortions still lags countries with earlier introduction. The percentage of abortions provided by medication continued to increase beyond the 31% previously reported in March 2020^15^ to over half of abortions in 2022. As countries with decades of mifepristone use have reached more than 70% to 90% abortions by medication (including increases following regulatory and service changes in response to the COVID-19 pandemic),^48,53^ we anticipate that this percentage will continue to increase over time.

Canada was well-positioned to seamlessly continue abortion service delivery throughout the COVID-19 pandemic,^23^ as prepandemic shifts from provision in purpose-specific or specialist abortion clinics to primary care following normally prescribed mifepristone^32^ facilitated pivots to telemedicine and remote abortion provision.^17,54^ In contrast, shifts to telemedicine or remote abortion provision occurred during the COVID-19 pandemic in other countries.^2,17,18,21,45,55^ However, contraception service provision (generally through primary care) was less consistent, leading to reduced access,^21,56^ especially for long-acting reversible contraceptives (which require in-person placement and removal). Shifts to telemedicine abortion and postabortion follow-up care may have reduced opportunities for postabortion intrauterine contraceptive device provision, potentially increasing repeat abortions.^33^ In this study, we disaggregated the possible influences of improved abortion access over this study period from instability in trends associated with the pandemic (with acute decreases and subsequent increase) from potential real changes that may have been due to sociocultural shifts in jurisdictions where abortion rate jumps have been found.

### Limitations

The following limitations should be considered when interpreting these results. Reasons underlying population abortion rates are complex and often multifactorial. In the context of declining birth rates in Canada^57^ and internationally,^58^ changes in abortion rates may reflect changes in pregnancy rate trends, pregnancy intention, and/or social shifts in managing unintended pregnancy. This study could not consider underlying trends in pregnancy intention, as pregnancy intention information is not available in population-based health administrative data. While measures such as the abortion ratio are sometimes used as a proxy for pregnancy intention,^53^ this measure is vulnerable to several biases, including conception date misalignment and failure to include miscarriage.

The abortion rate could be estimated with some error in population-based health administrative data. In our study, some individuals who received a mifepristone dispensation may have subsequently decided not to take the medication (and thus erroneously counted as having had a medication abortion). With the abortion episode approach that we used in this study,^26^ we would detect such instances when the individual opted for a procedural abortion after receiving a mifepristone dispensation (and would not count these twice as 2 separate events). In instances when a pregnancy continued after mifepristone dispensation (with pregnancy ending in a subsequent miscarriage or birth), we were not able to differentiate between medication abortion failure versus patient decision to not take the mifepristone and thus continue the pregnancy. As our team has previously reported, postabortion ongoing pregnancy continuing to delivery remained exceedingly rare among those who filled their mifepristone dispensation (<0.5%).^15^

In contrast, it is not clear whether abortion rate estimates from jurisdictions with recently available self-managed and/or telemedicine abortion may be vulnerable to abortion rate overestimation. Rates of nonuse after mifepristone dispensation from 5% to 13% have been reported in other jurisdictions.^47,59,60^ Furthermore, in settings in which there was a self-managed abortion with medications acquired through Women on Web (WoW), an international nonprofit organization that facilitates remote medical abortions, or in which there was an advanced provision of mifepristone (before an unintended pregnancy has occurred),^61^ reported rates may have been unreliable. Since remote abortion provision through services such as WoW is generally not captured in abortion surveillance statistics, settings that experience shifts to telemedicine provision within the health system may report erroneous increased rates (newly detecting the fraction of abortions previously provided by WoW), or if shifts to advance provision occur, reported rates may erroneously decline. For example, when the requirement for in-person clinic visits for medication abortion in the UK and Ireland was discontinued, this resulted in a subsequent decrease in abortion requests through WoW.^46^ In contrast, increased use of WoW was observed in countries where in-person visits were still mandated during the pandemic.^46^ In Canada, such services are used infrequently, as the health system supports similar services free of charge for provincially insured individuals.

## Conclusions

The findings of this cohort study suggest that with longstanding declines continuing through 2016, abortion rates began to gradually increase with mifepristone availability in 2017 in Ontario, Canada. Accounting for pandemic-related rate variability, substantial increases in abortion rates reported elsewhere from 2020 to 2023 did not occur in Ontario as of 2022, suggesting that Ontario’s health services environment and Canada’s regulatory and policy approach to preserving reproductive health services may have helped stabilize abortion rates. Future research is needed to understand how sociocultural changes affecting abortion service use elsewhere may be affecting contraception access and use and thus abortion rates in Canada.

## Notes

Supplement 1. **eTable 1.** Databases and Diagnostic, Procedure, Billing, and Other Codes Used to Create Abortion Episodes, Create Study Cohort, and Classify Abortion Method **eTable 2.** Model Transformations for Abortion Rate Trends **eTable 3.** Abortion Rates in Ontario, Canada, Over 4 Time Periods From January 2012 to December 2022 Overall and Within Age Categories

Supplement 2.
Data Sharing Statement
