# Supplementary material for: Trends in Abortion Rates in Ontario, Canada
Source: JAMA Netw Open. 2025 Apr 11;8(4):e254516. doi: 10.1001/jamanetworkopen.2025.4516 (PMC11992605; doi:10.1001/jamanetworkopen.2025.4516)
Supplement: Supplement 1. — eTable 1. Databases and Diagnostic, Procedure, Billing, and Other Codes Used to Create Abortion Episodes, Create Study Cohort, and Classify Abortion Method eTable 2. Model Transformations for Abortion Rate Trends eTable 3. Abortion Rates in Ontario, Canada, Over 4 Time Periods From January 2012 to December 2022 Overall and Within Age Categories [file jamanetwopen-e254516-s001.pdf]

Supplemental Online Content

Schummers L, Dunn S, Cheng L, Norman WV. Trends in abortion rates in Ontario, Canada. *JAMA Netw Open*. 2025;8(4):e254516. doi:10.1001/jamanetworkopen.2025.4516

eTable 1. Databases and Diagnostic, Procedure, Billing, and Other Codes Used to Create Abortion Episodes, Create Study Cohort, and Classify Abortion Method
eTable 2. Model Transformations for Abortion Rate Trends
eTable 3. Abortion Rates in Ontario, Canada, Over 4 Time Periods From January 2012 to December 2022 Overall and Within Age Categories

This supplemental material has been provided by the authors to give readers additional information about their work.

© 2025 Schummers L et al. *JAMA Network Open*.

eTable 1. Databases and Diagnostic, Procedure, Billing, and Other Codes Used to Create Abortion Episodes, Create Study Cohort, and Classify Abortion Method

	**Variable**	**Hospital data:**	**Billing data:**	**Other data sources**
Data set details		Discharge Abstract Database (DAD), Same Day Surgery (SDS), National Ambulatory Care Reporting System (NACRS) codes	Ontario Health Insurance Program (OHIP)	Ontario Drug Benefit database; MOMBABY linkage for birth records; Registered Persons Database
Inclusion				
	Medication abortion	CCI code 5CA88 accompanied by ICD-10 diagnostic code O04	Fee code A920, 895, or P001 accompanied by ICD-9 635	Ontario Drug Benefit database DIN 2444038 (mifepristone)
	Procedural abortion	CCI procedure code 5CA89 accompanied by ICD-10 diagnostic code O04	Fee code S752, S785	
	Unclassified abortion	CCI code 5CA20, 5CA24 accompanied by ICD-10 diagnostic code O04 with no additional abortion codes (that could be used to classify the method).		
Exclusions				
	Miscarriage/ectopic pregnancy codes appear in 42-day abortion episode window. Exception: if the first code of the episode is for an abortion and the only miscarriage/ectopic code that occurs in the episode is an ectopic pregnancy, classify the episode as abortion.	ICD-10-CA codes O00, O01, O02, O03, O05, O0800, O0810, O0820, O0830, O0840, O0850, O0860, O0870, O0880, O0890, CCI code 5CA93	Fee codes A922, S756, S768<br>ICD9 codes 632, 634, 637	
	Abortion event occurs within 42 days of a miscarriage/ectopic pregnancy code.	ICD-10-CA codes O00, O01, O02, O03, O05, O0800, O0810, O0820, O0830, O0840, O0850, O0860, O0870, O0880, O0890, CCI code 5CA93	Fee codes A922, S756, S768<br>ICD9 codes 632, 634, 637	
	Age range restriction			Registered Persons Database (RPDB) age <15 or ≥45

© 2025 Schummers L et al. *JAMA Network Open*.

	Non-resident of Ontario (no valid ON postal code on index abortion date)			Registered Persons Database (RPDB) no IKN
	Live at the time of abortion			Registered Persons Database (RPDB) death before index abortion date
	Female sex			Registered Persons Database (RPDB) male sex
	Record not included in yearly denominator cohort or ineligible for OHIP (found to not have OHIP coverage in all four quarters of index abortion year outside age range on Jul 1 of index abortion year; not found to have a valid Ontario postal code on Jul 1 of index abortion year)			Registered Persons Database (RPDB) no IKN, RPDBELIG=no
	Selective fetal reduction	CCI code 5CA90	Fee codes P053, P054	
	Within 42 days of a birth or delivery with gestational age ≥25 weeks			Record in MOMBABY
	Birth, stillbirth, or fetal demise code occurs within abortion episode			Record in MOMBABY
Create denominator cohort	• Pull yearly data from RPDB where date of last contact (DOLC) is within 7 years of January 1 of each respective year (2012-2022).<br>• Pull demographic information for July 1 of each respective year denominator pulled in step 1 and retain only Ontario residents in the 15-44 year age range.<br>• Check OHIP eligibility in each calendar year quarter and retain only records with OHIP eligibility in every quarter.			
Hierarchy to define abortion type	If two or more of the above events occurring on the same day, pick the top code in the following hierarchy:<br>• OHIP billing code S785 (procedural)<br>• OHIP billing code P001 accompanied by ICD-9 code 635 (medication)<br>• OHIP billing code P001 accompanied by ICD-9 code 895 (medication)<br>• OHIP billing code S752 (procedural)<br>• DAD/NACRS/SDS CCI code 5CA89 accompanied by ICD-10 code O04 (procedural)<br>• ODB DIN code 02444038 (medication)<br>• DAD/NACRS/SDS CCI code 5CA88 accompanied by ICD-10 code O04 (medication)<br>• OHIP billing code A920 accompanied by ICD-9 code 635 (medication)<br>• OHIP billing code A920 accompanied by ICD-9 code 895 (medication)<br>• DAD/NACRS/SDS CCI code 5CA20 accompanied by ICD-10 code O04 (undefined)<br>• DAD/NACRS/SDS CCI code 5CA24 accompanied by ICD-10 code O04 (undefined)			
	If an abortion code occurs within 6 weeks of another abortion code, use the following hierarchy to define index abortion event:<br>Episodes with ≥2 medication abortion codes<br>• If P001 occurs anywhere in the record, make it the index event.<br>• Else if 02444038 occurs in the record:<br>o If 02444038 occurs once in the record, make it the index event.			

© 2025 Schummers L et al. *JAMA Network Open*.

	○ If 02444038 occurs twice in the record and events are 1-6 days apart, use the second date as the index event (second event is ‘replacement dose’).<br>○ If 02444038 occurs twice in the record and events are 1-6 weeks apart, use the first date as the index event (second dose is complication, indicating need for further treatment).<br>• Else if 5CA88 occurs anywhere in the record, make it the index event.<br><br>Episodes with ≥2 procedural abortion codes<br>• If S785 appears in the second record and the records are 1-3 days apart, use S785 as the index event.<br>• Else use the first code as the index event.<br><br>Episodes with a medication abortion code followed by a procedural abortion code<br>• If a non-P001 medication abortion code occurs 1-6 days before a procedural code, define as a procedural abortion (rationale: patient changed mind about abortion method).<br>• If P001 occurs 1-3 days before a procedural abortion, define as medication; otherwise, define as procedural.<br>• If any medication abortion code occurs ≥7 days before a procedural abortion code, define as medication (procedural event is to manage incomplete medication abortion/complication, subsequent uterine aspiration event).<br><br>Episodes with a procedural abortion code followed by a medication abortion code<br>• If a procedural abortion code appears 1-3 days before medical abortion code P001, define as medication with P001 as index event (procedural abortion event is preparation for induction abortion).<br>• Else if a procedural abortion code occurs before a medication abortion code, define as procedural with procedural abortion date as index event.<br><br>Episodes with an undefined abortion code occurring first<br>• If a medication or procedural abortion code occurs within 6 weeks of the undefined abortion code, use the medication or procedural abortion code as the index event.<br>• Otherwise, use the undefined code.
Codes and hierarchy used to define miscarriage and ectopic pregnancy	If multiple miscarriage or ectopic pregnancy codes in the same episode, classify according the following hierarchy:<br>• Ectopic pregnancy<br>○ OHIP billing code A922<br>○ DAD/NACRS/SDS ICD-10 code O00, O0800, O0810, O0820, O0830, O0840, O0850, O0860, O0870, O0880, O0890, CCI code 5CA93<br>• Hydatidiform mole<br>○ DAD/NACRS/SDS ICD-10 code O01<br>• Spontaneous abortion<br>○ OHIP ICD-9 code 634<br>○ OHIP billing code S768<br>○ DAD/NACRS/SDS ICD-10 code O03<br>• Missed abortion<br>○ OHIP billing code S756<br>○ OHIP ICD-9 code 632<br>○ DAD/NACRS/SDS ICD-10 code O02.1<br>• Other<br>○ OHIP ICD-9 code 637<br>○ DAD/NACRS/SDS ICD-10 code O05<br>○ DAD/NACRS/SDS ICD-10 code O02

.

© 2025 Schummers L et al. *JAMA Network Open*.

eTable 2. Model Transformations for Abortion Rate Trends
i. pre-mifepristone (January 2012 – December 2016) and ii. after mifepristone availability as a normally prescribed medication and before the COVID-19 pandemic (October 2017 – March 2020)

	i. Pre-mifepristone	ii. After mifepristone availability as a normally prescribed medication and before the COVID-19 pandemic
	Jan 2012-Dec 2016	Oct 2017 – Mar 2020
Age 15-44	log	log
Age 15-19	log	log
Age 20-24	quadratic	linear
Age 25-29	log	log
Age 30-34	quadratic	linear
Age 35-44	log	log

© 2025 Schummers L et al. *JAMA Network Open*.

eTable 3. Abortion Rates in Ontario, Canada, Over 4 Time Periods From January 2012 to December 2022 Overall and Within Age Categories

i) Baseline (January – March 2012); ii) before mifepristone introduction (October – December, 2016); iii) After mifepristone availability as a normally prescribed medication and before the COVID-19 pandemic (January – March 2020), and iv) After the COVID-19 pandemic (October – December 2022).

	Baseline		Pre-mifepristone	Post-mifepristone, pre-pandemic		Post-pandemic		
	Number of abortions, Jan – Dec 2012	Rate (95% CI) Jan-Mar 2012	Rate (95% CI) Oct-Dec 2016	Observed rate (95% CI) Jan-Mar 2020	Expected rate (95% CI) Jan-Mar 2020	Crude observed rate Jan-Dec 2022	Expected rate based on pre-mifepristone trend (95% CI)	Expected rate based on post-mifepristone trend (95% CI)
Age 15-44	42,015	16.0 (15.5, 16.6)	13.6 (13.3, 14.0)	13.8 (13.5, 14.3)	12.3 (11.5, 13.5)	14.2	11.3 (9.8, 13.3)	13.2 (12.9, 16.1)
Age 15-19	5,142	13.8 (13.0, 14.4)	7.9 (7.5, 8.4)	7.8 (7.3, 8.3)	5.9 (4.6, 7.2)	7.0	4.4 (2.2, 6.5)	7.2 (5.8, 9.8)
Age 20-24	12,294	28.1 (27.3, 28.9)	21.5 (20.7, 22.3)	20.5 (19.7, 21.3)	18.0 (13.2, 22.2)	21.4	15.7 (10.5, 20.1)	19.9 (18.8, 21.3)
Age 25-29	10,055	23.1 (22.1, 24.1)	20.1 (19.5, 20.7)	19.8 (19.3, 20.6)	19.0 (17.3, 20.7)	20.8	18.2 (15.2, 21.0)	19.2 (17.5, 22.9)
Age 30-34	7,310	16.4 (16.3, 16.8)	15.7 (15.6, 16.1)	16.9 (16.5, 17.0)	14.0 (13.6, 16.2)	16.9	11.8 (10.9, 16.7)	17.9 (16.9, 18.4)
Age 35-44	7,214	7.5 (7.1, 7.9)	7.7 (7.5, 7.9)	8.5 (8.4, 8.9)	7.4 (6.7, 8.0)	8.9	7.1 (6.0, 8.1)	8.8 (8.0, 10.7)

Rates are adjusted for seasonality.
95% confidence intervals estimated using bootstrapping.

© 2025 Schummers L et al. *JAMA Network Open*.
